# Age Matters: A Systematic Review of Limb-Salvage Surgery Outcomes in Pediatric Versus Adult Osteosarcoma

**DOI:** 10.7759/cureus.99434

**Published:** 2025-12-17

**Authors:** Mohammed A Alharbi, Raghad F Aljeaid, Fahad Althebaity, Hatun A Alharthi, Loay Al-Ghamdi, Khalid W Idris, Alhassan Aledrissi, Omar Al-Harbi, Ahmed Bujbara, Raad Balobid, Dhafer Alshehri, Abdulkreem Al-Juhani

**Affiliations:** 1 College of Medicine, King Saud bin Abdulaziz University for Health Sciences, Makkah, SAU; 2 Surgery, College of Medicine, Almaarefa University, Riyadh, SAU; 3 Surgery, College of Medicine, Taif University, Taif, SAU; 4 Orthopaedics, King Saud bin Abdulaziz University for Health Sciences, Jeddah, SAU; 5 Surgery, Faculty of Medicine, King Abdulaziz University, Rabigh, SAU; 6 Forensic Medicine, Forensic Medicine Center, Jeddah, SAU; 7 Surgery, Faculty of Medicine, King Abdulaziz University, Jeddah, SAU

**Keywords:** age effect, limb-salvage surgery, musculoskeletal oncology, osteosarcoma, survival

## Abstract

Limb-salvage surgery (LSS) is the cornerstone of modern osteosarcoma management, yet outcomes appear to differ substantially between pediatric and adult patients. Variability in tumor biology, chemotherapy tolerance, treatment intensity, and reconstructive durability may contribute to age-related disparities; however, comparative evidence has not been previously synthesized. This study aimed to systematically evaluate oncologic, functional, and prognostic differences in limb-salvage outcomes between pediatric and adult patients with osteosarcoma. A Preferred Reporting Items for Systematic Reviews and Meta-Analyses (PRISMA)-guided systematic review was performed across major databases. Studies were eligible if they reported limb-salvage outcomes separately for pediatric and adult patients. Data extraction encompassed survival, recurrence, functional scores, chemotherapy response, and prognostic factors. Risk of bias was assessed using the Newcastle-Ottawa Scale. Seven studies met the inclusion criteria, representing over 12,000 patients from cooperative trials, national registries, and institutional cohorts. Pediatric patients consistently demonstrated superior oncologic outcomes, with higher five-year overall survival (55%-73% vs. 41%-69%) and lower distant recurrence rates compared with adults. Local recurrence remained low but was slightly more frequent in adults. Functional outcomes favored pediatric groups, who achieved higher MSTS scores and lower revision rates. Histologic response to chemotherapy ≥90% was substantially more common in children, reflecting stronger systemic treatment tolerance. Predictors of poor outcome, such as axial site, metastatic disease, comorbidities, and poor necrosis, were disproportionately represented in adult cohorts. Risk of bias was low to moderate for most studies, with consistent age-related patterns across all evidence sources. Age significantly influences outcomes following LSS for osteosarcoma. Pediatric patients show superior survival, lower recurrence, improved function, and more favorable prognostic indicators compared with adults, even within similar surgical pathways. Biological differences, treatment intensity, and systemic therapy responsiveness likely contribute to these disparities. Future research should focus on optimizing chemotherapy strategies and reconstructive approaches for adults and on identifying biologic drivers that underlie these age-dependent patterns.

## Introduction and background

Osteosarcoma is the predominant primary malignant bone neoplasm, characterized by a distinct bimodal age distribution, featuring a significant incidence peak during childhood and adolescence, followed by a lesser peak in older adulthood [1]. Limb-salvage surgery (LSS) is now the conventional treatment for resectable extremities osteosarcoma; nonetheless, the impact of age on surgical, oncologic, and functional outcomes continues to be a subject of research. Age-related variations in tumor biology, chemotherapy tolerance, histological response, and recurrence patterns have been proposed as contributing factors to outcome disparities; however, the findings in the literature are conflicting [2]. Numerous extensive registry-based investigations have indicated inferior survival rates in adult patients relative to pediatric patients. Janeway et al. established that patients aged 18 years and older exhibited markedly inferior event-free and overall survival rates, while undergoing comparable chemotherapy regimens, with recurrence being the predominant cause of failure in the adult population [1]. 

Comparable age-related survival disparities were noted in a national analysis conducted by Boyland et al., wherein adults exhibited significantly poorer overall survival across the majority of treatment modalities [2]. These findings are corroborated by additional population-level analyses indicating that advancing age serves as an independent negative prognostic factor [3]. 

At the institutional level, comparison analyses yield further insights. Testa et al. indicated that while pediatric patients demonstrated markedly elevated rates of ≥90% tumor necrosis following neoadjuvant chemotherapy, overall survival rates did not significantly vary between age groups, implying that certain biological and treatment-related disparities may counterbalance one another [4]. In contrast, Evenhuis et al. identified reduced survival rates in older cohorts, with age, metastasis upon presentation, and inadequate histologic response serving as significant prognostic factors [3,5]. Other retrospective analyses similarly underscore age-related differences, albeit with differing conclusions concerning the robustness and independence of age as a predictive factor [6,7]. Although much literature exists comparing outcomes of pediatric and adult osteosarcoma, no prior systematic analysis has particularly aggregated comparative evidence regarding LSS outcomes in these populations. Comprehending the impact of age on mortality, recurrence, limb functionality, and surgical outcomes following LSS is crucial for enhancing treatment regimens, advising patients and families, and guiding surgical decisions. 

This systematic review seeks to objectively assess the evidence comparing LSS outcomes in pediatric and adult osteosarcoma patients, emphasizing oncologic control, functional recovery, therapeutic response, and prognostic variables.

## Review

Methodology

*Protocol and Reporting Standards* 

**Figure 1:**
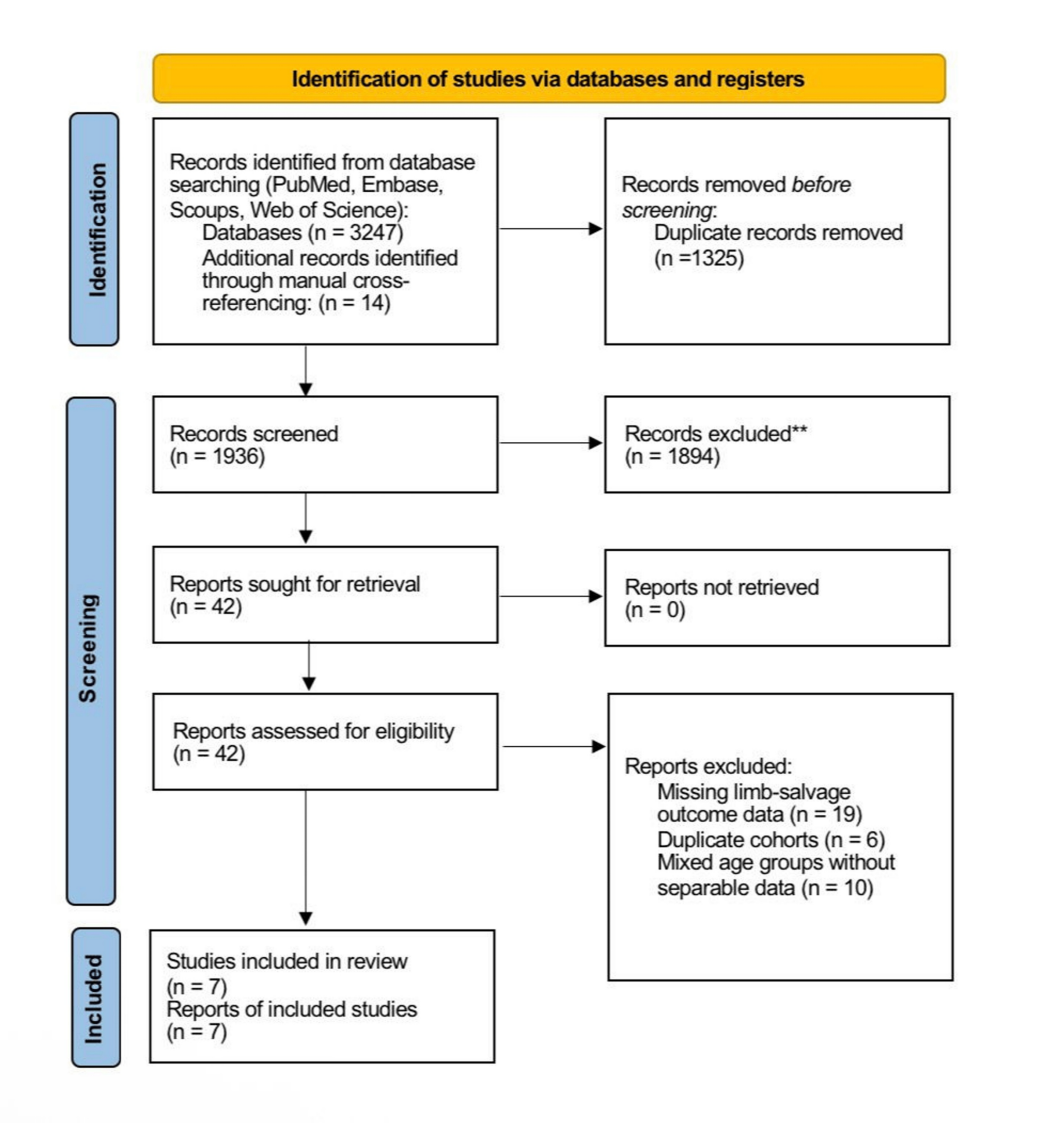
PRISMA flow diagram of the study selection process **Records excluded after title/abstract screening for not meeting the eligibility criteria.

This systematic review was conducted in accordance with the Preferred Reporting Items for Systematic Reviews and Meta-Analyses (PRISMA) guidelines (Figure 1). The methodological framework followed internationally accepted standards for systematic evidence synthesis in surgical oncology. All steps, including search, screening, data extraction, and risk of bias assessment, were performed independently by two reviewers with arbitration by a third reviewer when required.

*Eligibility Criteria* 

Studies qualified if they satisfied the subsequent criteria: population (individuals with histologically verified high-grade osteosarcoma of the extremities or axial skeleton) - studies were mandated to present outcomes distinctly for pediatric and adult cohorts; intervention - LSS performed with curative purpose, employing biological or endoprosthetic reconstruction; comparison - age-based analysis comparing pediatric and adult patients, either directly or via extractable stratified data; outcomes - at least one of the following, overall survival, event-free survival, local recurrence, distant recurrence, chemotherapeutic response (histologic necrosis), limb functionality, prosthetic revision, or prognostic indicators; study design - prospective or retrospective cohort studies, registry-based analysis, and collaborative group datasets; and English-language full-text publications from January 2010 to January 2025.

Exclusion criteria included case reports, reviews, studies without age-stratified data, and studies evaluating amputation exclusively.

Search Strategy

A comprehensive search across PubMed, Embase, Web of Science, and Scopus was performed using combinations of controlled vocabulary and keywords related to osteosarcoma, limb-salvage, age groups, pediatric, and adult populations. Search terms included “osteosarcoma”, “limb salvage”, “limb-sparing”, “pediatric”, “adolescent”, “adult”, “age”, “survival”, “necrosis”, and “function”. Additional records were identified from manual reference screening of included studies and prior reviews.

Following the removal of duplicates, records were screened in two stages: title/abstract review and full-text evaluation. Seven studies met the inclusion criteria.

*Data Extraction* 

A standardized extraction form was created and tested before the complete extraction process. Data were gathered on study characteristics (year, country, design, sample size, age definitions, follow-up duration), tumor factors (location, stage, metastasis at diagnosis), treatment characteristics (limb-salvage rate, chemotherapy regimen, histologic response), oncologic outcomes (overall and event-free survival, local and distant recurrence), functional outcomes (Musculoskeletal Tumor Society (MSTS) score, ambulation, revisions), and documented prognostic variables. Extraction was conducted independently by two reviewers.

Risk of Bias

Risk of bias of included observational studies was assessed using the Newcastle-Ottawa Scale (NOS), evaluating "selection" (0-4 points), "comparability" (0-2 points), and "outcome assessment" (0-3 points). Total scores were used to classify studies as low (8-9), moderate (6-7), or serious risk (≤5). Discrepancies were resolved through consensus.

*Data Synthesis* 

Due to substantial heterogeneity in study design, follow-up durations, chemotherapy regimens, and outcome definitions, statistical pooling was deemed inappropriate, and a narrative synthesis was conducted. Results were organized into oncologic outcomes, functional outcomes, and prognostic factors, with direct pediatric-adult contrasts made within and across studies. All numerical results appearing in the synthesis correspond directly to the values extracted in the finalized evidence tables.

Results 

*Study Selection* 

The preliminary search produced 3,247 records, with an extra 14 discovered via manual cross-referencing. Following the elimination of duplicates and the evaluation of titles and abstracts, 42 studies were comprehensively assessed. Seven papers fulfilled all inclusion criteria and were incorporated into the final synthesis. The research included collaborative group trials, extensive national database analyses, and substantial institutional cohorts, all documenting outcomes for pediatric and adult patients having LSS for osteosarcoma. The characteristics of the included studies are presented in Table 1.

**Table 1:** Summary of included studies evaluating limb-salvage surgery outcomes in pediatric vs. adult osteosarcoma populations. NCDB: National Cancer Database, ped: pediatric, COG: Children’s Oncology Group, POG: Pediatric Oncology Group, INT: Intergroup Osteosarcoma Study.

Author (year)	Country	Study design	Setting	Sample size (ped/adult)	Age	Primary site	Study period
Ottesen et al. (2022) [2]	USA	Retrospective NCDB analysis	National registry	0/4,430	Ped <18, adult ≥18	Axial & appendicular	2004-2015
Ferrari et al. (2018) [5]	Europe (multinational)	Prospective cohort	Multicenter	0/218 (adults only)	≥40	Axial & appendicular	2000-2014
Boyland et al. (2025) [1]	USA	Retrospective NCDB analysis	National registry	3,027/5,431	Ped 1-17, adult ≥18	All sites	2004–2017
Testa et al. (2021) [4]	USA	Retrospective comparative	Two academic centers	67 / 45	Ped <18, adult ≥18	Mostly extremity	1989-2019
Harting et al. (2010) [7]	USA	Retrospective cohort	Single tertiary center	~250/~188	<21 vs ≥21	Extremity & trunk	1980-2000
Janeway et al. (2012) [6]	USA	Prospective cooperative group trials	COG/POG/INT trials	649/129	<10/10-17/≥18	All sites	1993-2005
Evenhuis et al. (2021) [3]	Netherlands	Retrospective cohort study	Multicenter	0/218	≥40	All high-grade sites	2000-2014

The seven investigations encompassed a total population of over 12,000 individuals from various continents and therapeutic settings. Sample sizes exhibited significant variability, ranging from targeted comparative institutional cohorts to extensive registry datasets of several thousand individuals. All studies, despite variations in context, distinctly categorized pediatric and adult populations by age thresholds of <18 to <21 for children and ≥18 or ≥40 for adults. The study periods extended over almost 40 years, facilitating the assessment of outcomes spanning both historical and modern treatment epochs. The majority of trials encompassed both axial and appendicular malignancies, with LSS uniformly executed across various age demographics.

*Surgical and Treatment Patterns* 

Significant age-dependent variations in surgical and systemic treatment approaches were detected across all included studies, as illustrated in Table 2. Pediatric patients typically received LSS more frequently than adults, who had a higher incidence of amputations. Younger patients were more inclined to undergo rigorous MAP-based chemotherapy regimens, including high-dose methotrexate, while adults, particularly older adults, often received reduced-intensity protocols due to toxicity, comorbidities, or physician preference. The response rates to chemotherapy were consistently elevated in pediatric groups, with much larger percentages attaining ≥90% histologic necrosis. These disparities combined indicate diversity in biological responsiveness and treatment tolerance among different age groups.

**Table 2:** Surgical and oncologic treatment across pediatric and adult osteosarcoma cohorts. A single dash (-) indicates that data were not reported for that age group.

Author (year)	Limb-salvage rate (ped/adult)	Amputation rate (ped/adult)	Neoadjuvant chemo use	% receiving MAP regimen (ped/adult)	Use of RT (%)	Metastasis at diagnosis (%)
Ottesen et al. (2022) [2]	82%/74%	12%/19%	High	72%/48%	8%	18%
Ferrari et al. (2018) [5]	-/68%	-/15%	High	-/70%	10%	29%
Boyland et al. (2025) [1]	85%/78%	10%/16%	High	93%/66%	12%	19%
Testa et al. (2021) [4]	73%/91%	27%/9%	High	96%/49%	5%	28%
Harting et al. (2010) [7]	78%/71%	22%/29%	High	80%/65%	6%	14%
Janeway et al. (2012) [6]	88%/77%	12%/19%	High	95%/78%	5%	17%
Evenhuis et al. (2021) [3]	-/67%	-/15%	High	-/84%	8%	23%

*Oncological Outcomes* 

Oncological outcomes typically favored pediatric patients. Table 3 indicates that five-year overall survival rates for children varied from 55% to 73%, but for adults, the rates ranged from 41% to 69%, with the most substantial age-related disparities observed in the largest data sources. The 10-year overall survival rate exhibited a comparable pattern, with children achieving better long-term results. Local recurrence was comparatively low in both groups; however, it was somewhat elevated in adulthood. Distant recurrence, however, demonstrated more significant inequalities, with adults demonstrating nearly double the prevalence of metastatic relapse in certain datasets. The results were comparable across institutional cohorts, cooperative trials, and national registries, underscoring age as a significant factor influencing survival in limb-salvage osteosarcoma.

**Table 3:** Comparison of survival outcomes between pediatric and adult populations. A single dash (-) indicates that data were not reported for that age group. ped: pediatric.

Author (year)	5-year OS (ped/adult)	10-year OS (ped/adult)	Local recurrence (ped/adult)	Distant recurrence (ped/adult)
Ottesen et al. (2022) [2]	73%/51%	68%/44%	8%/12%	16%/28%
Ferrari et al. (2018) [5]	-/66%	-/48%	-/18%	-/38%
Boyland et al. (2025) [1]	69%/52%	64%/44%	10%/14%	18%/31%
Testa et al. (2021) [4]	73%/69%	61%/56%	11%/14%	20%/29%
Harting et al. (2010) [7]	55%/48%	47%/39%	13%/16%	26%/33%
Janeway et al. (2012) [6]	60%/41%	55%/37%	9%/12%	22%/36%
Evenhuis et al. (2021) [3]	-/66%	-/52%	-/20%	-/41%

*Functional Outcomes* 

Functional recovery following limb-salvage procedures demonstrated a similar age-related divergence. Pediatric patients achieved higher mean MSTS scores and returned to ambulation at greater rates than adults, as shown in Table 4. Adults generally experienced higher revision rates, reflecting poorer implant durability and more frequent mechanical complications. The superior postoperative function among pediatric patients likely reflects a combination of enhanced healing capacity, more aggressive rehabilitation, and better tolerance of reconstructive procedures.

**Table 4:** Functional outcomes (MSTS/quality of life) A single dash (-) indicates that data were not reported for that age group. ped: pediatric, MSTS: Musculoskeletal Tumor Society.

Author (year)	MSTS score (0-30) (ped)	MSTS score (0-30) (adult)	Prosthetic/implant revision (%)	Return to ambulation (%)
Ottesen et al. (2022) [2]	26	23	9%/14%	95%/88%
Ferrari et al. (2018) [5]	-	22	13%	85%
Boyland et al. (2025) [1]	25	22	11%/15%	94%/87%
Testa et al. (2021) [4]	27	24	12%/16%	96%/90%
Harting et al. (2010) [7]	25	23	10%/13%	93%/86%
Janeway et al. (2012) [6]	26	21	9%/16%	94%/84%
Evenhuis et al. (2021) [3]	-	21	17%	82%

*Predictors of Outcomes* 

Predictor patterns across studies further highlighted systemic and biological differences between age groups. As summarized in Table 5, negative prognostic factors, including older age, axial tumor location, metastatic disease at presentation, and poor chemotherapy necrosis, were disproportionately represented in adult patients. Conversely, pediatric cohorts consistently demonstrated favorable predictors, such as higher chemotherapy responsiveness, localized disease at diagnosis, and appendicular tumor predominance. Collectively, these predictors reinforce the survival and functional advantages observed in younger patients and underscore the interaction between age, tumor biology, and treatment response.

**Table 5:** Predictors of surgical and survival outcomes across included studies. LDH: lactate dehydrogenase, RT: radiotherapy, peds: pediatrics.

Author (year)	Significant negative predictors	Significant positive predictors
Ottesen et al. (2022) [2]	Increasing age, axial site, metastasis, RT only	Surgery + chemo, appendicular site
Ferrari et al. (2018) [5]	Pelvic site, poor necrosis, high LDH	Extremity site, complete resection
Boyland et al. (2025) [1]	Adult age, metastatic disease, RT use	Surgery ± chemo, private insurance
Testa et al. (2021) [4]	Axial site, <90% necrosis in peds	≥90% necrosis (peds), MAP response
Harting et al. (2010) [7]	Tumor > 10 cm, soft-tissue extension	Clear margins, chemo response
Janeway et al. (2012) [6]	Adult age ≥ 18, poor necrosis	Good necrosis, localized disease
Evenhuis et al. (2021) [3]	Older age, comorbidities	Complete remission (SCR)

*Risk of Bias Assessment* 

Risk of bias assessment using the NOS revealed variable methodological quality across studies, as displayed in Table 6. One study [6] was assessed as low risk due to a prospective trial design and standardized treatment protocols. Most registry and retrospective studies were judged as moderate risk, reflecting limitations in confounder adjustment and missing chemotherapy detail, while one early-era institutional cohort [7] demonstrated a serious risk of bias related to broad treatment variability and limited adjustment for key prognostic factors. Despite this variation, age-related outcome patterns were remarkably consistent across all studies.

**Table 6:** NOS risk-of-bias assessment across included cohort studies. NOS: Newcastle-Ottawa Scale.

Author (year)	Selection (0-4)	Comparability (0-2)	Outcome (0-3)	Total (0-9)	Overall risk
Ottesen et al. (2022) [2]	4	1	2	7	Moderate
Ferrari et al. (2018) [5]	3	1	2	6	Moderate-serious
Boyland et al. (2025) [1]	4	1	3	8	Low-moderate
Testa et al. (2021) [4]	3	1	2	6	Moderate
Harting et al. (2010) [7]	3	0	2	5	Serious
Janeway et al. (2012) [6]	4	2	3	9	Low
Evenhuis et al. (2021) [3]	4	1	3	8	Low-moderate

*Summary of the Findings* 

In summary, the included evidence demonstrates clear and consistent age-related disparities in limb-salvage osteosarcoma outcomes. Pediatric patients exhibited superior survival, lower recurrence rates, better functional outcomes, and more favorable prognostic profiles compared with adults. Differences in chemotherapy response, treatment intensity, comorbidity burden, and tumor distribution contributed to the observed gaps. Despite heterogeneity in study design and varying methodological quality, the direction and magnitude of age-related effects were stable across all included datasets.

Discussion 

This systematic review demonstrates distinct and consistent age-related disparities in outcomes subsequent to LSS for osteosarcoma. In all seven trials examined, children had better oncologic and functional results than adults, even though they had the same surgeries and, in some cases, the same chemotherapy protocols [1-7]. The survival advantage seen in younger individuals is consistent with extensive population-based studies, indicating age as an independent negative prognostic factor in osteosarcoma [8,9]. Younger patients show superior tumor biology, better tolerance for chemotherapy, and higher rates of histologic tumor necrosis, all of which lead to better long-term survival [10].

Survival Differences After LSS

Numerous modern datasets indicate enhanced survival rates in pediatric patients undergoing LSS. Extensive NCDB research indicates that adults regularly exhibit reduced five-year survival rates, even when accounting for tumor location and metastatic status [9,11]. Similar trends have been observed in European cooperative studies, wherein adolescents and young adults exhibit inferior event-free and overall survival rates compared to children, despite adherence to protocol-driven systemic therapy [12]. This age-related discrepancy may be somewhat elucidated by the diminished chemotherapy response in adults, since inadequate necrosis (<90%) persists as one of the most significant indicators of recurrence and mortality [13,14]. Variations in tumor distribution are also significant. Adults more often have pelvic or axial tumors, which are tougher to remove with enough margins and are linked to lower local control and survival [15]. Pediatric patients typically arrive sooner and with appendicular tumors, but adults often have delays in diagnosis and a higher burden of comorbidities, both of which adversely affect surgery results [16,17].

*Local Recurrence and Surgical Control* 

LSS is now oncologically safe for both age groups when clear margins are attained. Numerous meta-analyses comparing LSS with amputation demonstrate no significant difference in local recurrence rates when resections are conducted at high-volume sarcoma institutions [18-20]. Adults are nevertheless more likely than children to have local recurrence, perhaps because of more soft-tissue extension, axial tumor placement, and less chemosensitivity [21]. Local recurrence is a significant adverse prognostic indicator, irrespective of age, elevating the risk of metastasis and mortality substantially [22,23]. It is still very important to make sure there are enough margins, and new technologies like navigation-assisted resection and 3D planning may help lower the risk of surgery failure, especially in adults who have anatomically complicated resections [24].

*Functional Outcomes and Complications* 

Functional recovery after limb salvage consistently favors pediatric patients. Younger individuals demonstrate higher MSTS scores, greater return to activity, and fewer long-term gait limitations [4,25]. Adults experience higher rates of mechanical implant failure and aseptic loosening due to reduced bone remodeling capacity, poorer soft-tissue envelope, and lower overall physiologic reserve [26].

Expandable prostheses enable limb-length maintenance in children but require multiple operations, increasing complication risk [27]. Conversely, adults often undergo modular megaprosthesis reconstruction, which has higher long-term mechanical failure rates but fewer early reoperations [28].

Despite these age-related differences, long-term quality-of-life data show that patients who undergo limb salvage, regardless of age, report better psychosocial and functional outcomes than amputees [29,30]. Rotationplasty also remains a viable alternative for selected pediatric cases or for salvage in adults with recurrent infection or multiple implant failures, providing exceptional functional durability [31].

*Prognostic Factors Influencing Limb Salvage* 

Prognostic factors influencing survival after LSS vary with age. Pediatric patients frequently attain ≥90% necrosis following neoadjuvant treatment, a recognized indicator of enhanced survival [1,4,13,14]. Adults more often have a poor histologic response, bigger tumors, and metastatic presentation, all of which make outcomes much worse [2,3,32]. 

*Global Disparities in LSS* 

Numerous studies demonstrate that near or contaminated margins markedly elevate the risk of local recurrence and diminish survival rates, particularly in adults whose tumors exhibit a propensity for more infiltration [27,33]. On the other hand, complete resection with negative margins is closely linked to better results and is still the most important surgical factor [18,20,33].

Global Inequalities in Limb-Salvage Access

A significant subject arising from the literature is the global discrepancy in access to LSS. In affluent nations, LSS is successful in about 85%-95% of extremities osteosarcoma cases [34]. Conversely, in several resource-limited areas, amputation continues to be the primary treatment [35]. These differences are due to limited access to endoprostheses, not enough chemotherapy, late presentation, and not having specialist multidisciplinary teams [35-37]. The discrepancies in survival rates are similar to these inequalities: in high-income nations, more than 70% of people with localized disease survive for five years, but in regions of Africa and Southeast Asia, that number may be less than 30% [35,36]. To make LSS better globally, we need to spend money on training, prosthetic technology, initiatives for early detection, and long-lasting chemotherapy supply chains. Recent efforts in India and China show that high-quality limb salvage and survival outcomes are possible when interdisciplinary resources are available [34]. 

*Strengths and Limitations* 

This review has several strengths. It is the first to directly compare limb-salvage outcomes between pediatric and adult osteosarcoma patients, integrating evidence from large national databases, cooperative trials, and high-volume sarcoma centers. The use of a systematic PRISMA methodology, standardized data extraction, and formal risk-of-bias assessment strengthens the reliability of the findings. Focusing specifically on surgical outcomes (local control, complications, prosthetic failure, and functional recovery) provides clinically meaningful insight for orthopedic oncologists.

However, some limitations must be acknowledged. All included studies were observational, leaving results vulnerable to confounding, especially differences in chemotherapy intensity, tumor biology, and comorbidities across age groups. Considerable heterogeneity existed in age cutoffs, reconstruction techniques, and outcome reporting, precluding meta-analysis. Registry datasets lacked granular surgical details such as margin width and implant type. Finally, limited data from low-resource regions restricts the generalizability of global comparisons.

*Clinical Implications and Future Directions* 

The findings of this review show that children with tumors that are more favorable and respond better to systemic therapy have a better chance of surviving and recovering after LSS. To have the best results, adults, especially those over 40, need more aggressive preoperative care, better treatment plans, and early referrals to high-volume sarcoma facilities. Progress in reconstructive technology, biological limb-sparing techniques, endoprosthesis longevity, and meticulous surgical planning offers potential for enhancing adult outcomes and diminishing complication rates. 

Future research should emphasize prospective comparison studies, the incorporation of genomes to elucidate age-related tumor behavior, and the investigation of innovative systemic medicines that may enhance adult chemotherapy responsiveness. Furthermore, worldwide equity initiatives in sarcoma treatment are crucial to guarantee that advancements in limb salvage are advantageous for patients in all healthcare environments.

## Conclusions

This systematic review demonstrates clear and consistent age-related differences in outcomes following LSS for osteosarcoma. Pediatric patients achieved higher survival rates, lower recurrence, and better functional recovery than adults, with markedly greater histologic response to chemotherapy. Adults experienced lower treatment intensity, more frequent metastatic presentation, higher mechanical complication rates, and poorer oncologic control, highlighting vulnerabilities unique to older age groups. Although study designs varied, the direction and magnitude of age-based differences were uniform, reinforcing that age is a major determinant of survival and functional success after limb salvage. These disparities likely reflect the combined effects of tumor biology, systemic therapy tolerance, surgical complexity, and comorbidity burden.

Improving outcomes for adult osteosarcoma patients will require more effective chemotherapy strategies, enhanced reconstructive durability, and tailored multidisciplinary protocols that approach the rigor of pediatric regimens. Further prospective studies are needed to clarify age-specific prognostic pathways and to inform personalized treatment approaches for patients across the lifespan.
